# The role of astrocytes from synaptic to non-synaptic plasticity

**DOI:** 10.3389/fncel.2024.1477985

**Published:** 2024-10-18

**Authors:** Rafael Sanz-Gálvez, Dominic Falardeau, Arlette Kolta, Yanis Inglebert

**Affiliations:** ^1^Department of Neurosciences, Université de Montréal, Montréal, QC, Canada; ^2^Centre Interdisciplinaire de Recherche sur le Cerveau et l’Apprentissage (CIRCA), Montréal, QC, Canada; ^3^Department of Stomatology, Université de Montréal, Montréal, QC, Canada

**Keywords:** astrocytes, synaptic plasticity, STDP, neuronal excitability, axonal plasticity

## Abstract

Information storage and transfer in the brain require a high computational power. Neuronal network display various local or global mechanisms to allow information storage and transfer in the brain. From synaptic to intrinsic plasticity, the rules of input–output function modulation have been well characterized in neurons. In the past years, astrocytes have been suggested to increase the computational power of the brain and we are only just starting to uncover their role in information processing. Astrocytes maintain a close bidirectional communication with neurons to modify neuronal network excitability, transmission, axonal conduction, and plasticity through various mechanisms including the release of gliotransmitters or local ion homeostasis. Astrocytes have been significantly studied in the context of long-term or short-term synaptic plasticity, but this is not the only mechanism involved in memory formation. Plasticity of intrinsic neuronal excitability also participates in memory storage through regulation of voltage-gated ion channels or axonal morphological changes. Yet, the contribution of astrocytes to these other forms of non-synaptic plasticity remains to be investigated. In this review, we summarized the recent advances on the role of astrocytes in different forms of plasticity and discuss new directions and ideas to be explored regarding astrocytes-neuronal communication and regulation of plasticity.

## Introduction

The rules governing changes in synaptic and intrinsic plasticity are diverse and complex, sometimes synergistic and sometimes not (Debanne et al., 2019). Most studies have been neuro-centric, despite growing evidence that astrocytes can intervene or interact to modify or modulate synaptic transmission (Araque et al., 1998; Jourdain et al., 2007; Bonansco et al., 2011), input integration, neuronal excitability (Tan et al., 2017), spike waveform or axonal conductivity (Sasaki et al., 2011; Lezmy et al., 2021). Astrocytes can detect neuronal activity, and depending on the firing rate of action potentials (APs), they can not only release gliotransmitters such as adenosine or glutamate (Hamilton et al., 2008; Lezmy et al., 2021), but also trigger intracellular calcium ([Ca^2+^]_i_) oscillations at different frequencies (Pasti et al., 1997). Thanks to recent technical advances these [Ca^2+^]_i_ responses can now be measured with great precision, such as in the recent approach published by the group of Volterra allowing for 3D calcium imaging (Savtchouk et al., 2018). In this review, we focus on the forms of synaptic and intrinsic plasticity that have not received much attention in relation to astrocyte involvement. Foremost, there is a more physiological form of synaptic plasticity known as spike timing-dependent plasticity (STDP), demonstrated both *in vivo* and *in vitro* across a range of species from rodents to humans. STDP is particularly relevant because it is believed to underlie spatial and episodic memory in the hippocampus. STDP is known to be affected by factors such as neuromodulation (Brzosko et al., 2019), extracellular calcium levels (Inglebert et al., 2020; Inglebert and Debanne, 2021), and activity patterns, all of which can be influenced by astrocytes through various mechanisms discussed below. Since synaptic plasticity is not the only mechanism involved in memory formation, we also discuss the often-overlooked potential role of astrocytes in intrinsic excitability. Plasticity of intrinsic excitability can act through numerous mechanisms such as voltage-gated ion channels (VGIC) regulation or the plasticity of excitable axonal domains. Synaptic and intrinsic plasticity are closely related, as changes in one will inevitably impact the other.

## Astrocytes and spike timing-dependent plasticity

**FIGURE 1:**
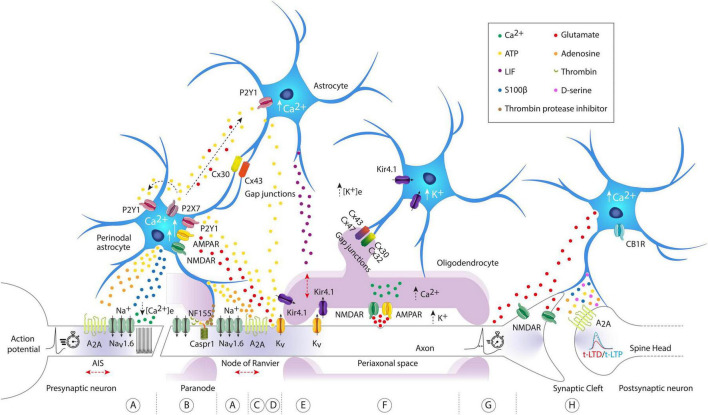
Molecular architecture of axon-astrocyte-oligodendrocyte communication pathways regulating intrinsic excitability and synaptic plasticity. **(A)** Astrocyte activated by arrival of action potentials (APs), can release ATP/adenosine to activate A_2A_ axonal receptors, regulating both neuronal excitability at the axon initial segment (AIS) and the conduction velocity (CV) of the AP at the nodes of Ranvier. They can also release S100β, decrease the concentration of extracellular calcium ([Ca^2+^]_e_), and modify neuronal firing patterns. **(B)** Through the vesicular release of protease thrombin inhibitors, perinodal astrocytes can regulate the binding of myelin to the axon, precisely at the binding site of the cell adhesion molecule NF155 and Caspr1, interfering with the detachment of the outer paranodal loops and the widening of the nodal space. The widening of the nodal space causes lateral diffusion of voltage-gated Na^+^ channels and a slowing of AP CV. **(C,D)** Astrocytic [Ca^2+^]_i_ signaling is evoked by axonal release of ATP and glutamate driven by APs. Axonal glutamate evokes small astroglial Ca^2+^ signals directly by acting on NMDA and AMPA receptors (short arrow). In contrast, axonal ATP induces large Ca^2+^ signals in astrocytes (long arrow), further stimulating the release of ATP and glutamate, which act as gliotransmitters in an autocrine and paracrine manner, propagating the signal to neighboring astrocytes. Note that astrocytes are highly interconnected through gap junction channels composed of connexin 30 (Cx30) and connexin 43 (Cx43). Axonal ATP release induces astrocytic secretion of LIF, which subsequently enhances myelination. **(E,F)** Oligodendrocytes can detect and respond to axonal activity either through axonal K^+^ release or through vesicular axonal glutamate release. In the first case, the axonal K^+^ influx pathway exits from the juxtaparanodal axolemma to be transported to the astrocytic syncytium, first through oligodendroglial Kir4.1 channels, then crossing successive paranodal loops, and finally reaching through astrocyte/oligodendrocyte gap junctions. Note that the uptake of K^+^ released by the axons is mostly performed by astroglial Kir4.1 channels. In the second case, APs can induce vesicular release of axonal glutamate, activating internodal NMDA and AMPA receptors, leading to an increase in [Ca^2+^]_i_ in oligodendrocytes. **(G)** The release of astroglial glutamate can modify the AP waveform. **(H)** Astrocytes exhibit elevated [Ca^2+^]_i_ levels through activation of CB1R. This calcium increase triggers glutamate release, which then activates presynaptic NMDA receptors to induce t-LTD (time-dependent long-term depression). Note that astrocytes can also spontaneously release glutamate and induce slow inward currents (SICs) in adjacent neurons, mediated by extrasynaptic NMDA receptors. Other gliotransmitters such as D-serine, ATP, adenosine, and S100β have been illustrated for their impact and modulation of synaptic plasticity. Observe that the red arrows indicate potential structural changes in excitable axonal domains. AIS, axon initial segment; ATP, adenosine triphosphate; A_2A_, adenosine A_2a_ receptor; NF155, neurofascin 155; Caspr1, contactin-associated protein-1; Kir4.1, inward rectifier potassium channels; CB1R, cannabinoid receptor-1; LIF, leukemia inhibitory factor; P2Y1-P2X7, two types of purinergic receptors.

Spike timing-dependent plasticity is a form of synaptic plasticity where changes in synaptic strength are influenced by the precise timing of activity between pre- and post-synaptic neurons. Generally, when pre-synaptic activity precedes post-synaptic activity, it results in long-term potentiation (t-LTP). Conversely, when post-synaptic activity comes before pre-synaptic activity, it leads to long-term depression (t-LTD). Only a few studies, listed in Table 1, have begun exploring the role of astrocytes in this process. One pioneering study examining inputs from layer 4 to layer 2/3 (L4-2/3) in the barrel cortex demonstrated that astrocytes are required for t-LTD (Min and Nevian, 2012). During t-LTD induction, the level of intracellular calcium increases in astrocytes due to activation of cannabinoid-1 receptors (CB1Rs). This calcium increase prompts the release of glutamate, which then activates presynaptic NMDA receptors (pre-NMDARs) to induce t-LTD (see Figure 1H). In addition, electrical stimulation of astrocytes with a series of depolarizing pulses alone can induce long-term depression (LTD) in nearby pyramidal neurons. Interestingly, at corticostriatal synapses, STDP is controlled by endocannabinoid (eCB) levels (Cui et al., 2016). One might speculate whether astrocytic CB1Rs are also implicated in this process. Following this study, astrocytes’ involvement in STDP through pre-NMDARs has been reported in other brain regions. At hippocampal CA3-CA1 synapses, a developmental switch from t-LTD to a presynaptic form of t-LTP involving adenosine and glutamate release from astrocytes was shown to occur (Falcón-Moya et al., 2020; see Figure 1H). A similar observation has been recently made at L4-2/3 synapses in the somatosensory cortex (Martínez-Gallego et al., 2022). Interestingly, the loss of adenosine receptors is associated with lack of t-LTD in the hippocampus (Pérez-Rodríguez et al., 2019), highlighting the importance of adenosine in synaptic plasticity, which has been recently reviewed here (Martínez-Gallego and Rodríguez-Moreno, 2024). The role of astrocytes in glutamate clearance has also been shown to be essential for STDP. In the striatum, blockade of the astrocytic excitatory amino-acid transporter type-2 (EAAT2) leads to a form of timing-independent LTP while its overexpression prevents any plasticity from occurring (Valtcheva and Venance, 2016). Despite these recent advances, the role of astrocytes in STDP at most of the synapses is still overlooked. For example, a recent study has identified two new forms of t-LTD at synapses between the entorhinal cortex and dentate gyrus, both of which require astrocyte activity (Cuaya et al., 2024). In addition, in the hippocampus, spontaneous glutamate release from astrocytes has been proposed to control the threshold of t-LTP (Bonansco et al., 2011). Noteworthy, specialized hippocampal astrocytes able to rapidly (on the subsecond scale) release glutamate at precise hotspots have been recently identified (De Ceglia et al., 2023). STDP has been shown to exist at the scale of the single spine (Tazerart et al., 2020) and astrocytes could easily be involved in the local control of plasticity at the level of a single synapse. This calls to revisit past work in the light of this recent evidence. Moving forward, it is crucial to always take into account the potential role of astrocytes while aiming to develop a proper plasticitome (Sjöström, 2021). In addition, all the work mentioned previously (and summarized in Table 1) has been focused on excitatory to excitatory synapses, but astrocytes are known to respond to inhibitory activity (Perea et al., 2016). STDP exists in different forms depending on the synapse considered (Feldman, 2012) and astrocytes participation will likely be different as well.

**TABLE 1:** Astrocytes and spike timing-dependent plasticity.

References	Region	Synapse	Preparation	Protocol	Astrocytes implication
Min and Nevian, 2012	Barrel cortex	L4-L2/3	Acute slices, rats, P16-21	Δt = −25 ms, 60 pairings at 0.2 Hz	Glutamate releases from astrocytes following activation of CB1R is necessary for t-LTD
Falcón-Moya et al., 2020	Hippocampus	CA3-CA1	Acute slices, mice, P13-21, P22-30, or P35-42	Δt = −18 ms, 100 pairings at 0.2 Hz	Glutamate and adenosine release from astrocytes is required for a development switch from t-LTD to t-LTP
Martínez-Gallego et al., 2022	Somatosensory cortex	L4-L2/3	Acute slices, mice, P13-27 or P38-P60	Δt = −18 ms, 100 pairings at 0.2 Hz	Glutamate and adenosine release from astrocytes is required for a development switch from t-LTD to t-LTP
Pérez-Rodríguez et al., 2019	Hippocampus	CA3-CA1 synapses	Acute slices, mice, P13-18 or P22-P30	Δt = −18 ms, 100 pairings at 0.2 Hz	Adenosine release from astrocytes is required for the developmental loss of t-LTD
Cuaya et al., 2024	Cortex/hippocampus	LPP- or MPP-GC synapses	Acute slices, mice, P13-21	Δt = −18 ms, 100 pairings at 0.2 Hz	Astrocytes signaling and glutamate release from astrocytes are required for t-LTD
Bonansco et al., 2011	Hippocampus	CA3-CA1 synapses	Acute slices, rats, P25-35	Δt = +10 ms, 60 or 120 pairings at 1 Hz	Spontaneous glutamate release from astrocytes controls the threshold of t-LTP

This table summarizes the role of astrocytes during synaptic plasticity induced by spike timing-dependent plasticity paradigm. The region, synapses, type of preparation, and protocol are indicated. In particular, the timing, the number of repetitions (pairings) and frequency is indicated as it is especially important for STDP. For example, Δt = −25 ms, 60 pairings at 0.2 Hz means that the EPSP-AP pairing is repeated 60 times at 0.2 Hz with a delay of 25 ms between the EPSP and the AP. CB1R, cannabinoid-1 receptor; t-LTD/LTP, timing LTD/LTP, respectively; LPP/MPP, lateral or medial perforant pathways, respectively; GC, granule cells.

## Astrocytes ion homeostasis on neuronal activity

Synaptic plasticity (including t-LTP and t-LTD) require post-synaptic calcium elevation to occur (Lisman, 1989; Nevian and Sakmann, 2006). One important function of astrocytes is to regulate local ion homeostasis such as extracellular calcium ([Ca^2+^]_e_) or potassium ([K^+^]_e_). Astrocytes can tightly control extracellular Ca^2+^ through S100β release (Morquette et al., 2015). S100β is often used as a specific astrocytic marker (Zhang et al., 2019) but is also a calcium-binding protein (Hermann et al., 2012). For example, optogenetic activation of astrocytes in layer 5 of the visual cortex has been shown to decrease [Ca2+]e specifically through the release of S100β (Ryczko et al., 2021). Interestingly, [Ca^2+^]_*e*_ has been shown to be critical for normal expression of LTP and LTD in the hippocampus and the cortex (Forsberg et al., 2019; Inglebert et al., 2020; Inglebert and Debanne, 2021; Chindemi et al., 2022). At CA3-CA1 synapses, reduction of [Ca^2+^]_*e*_ to physiological levels (1.3–1.8 mM) can prevent t-LTP, promote t-LTD, or even block the induction of plasticity (Inglebert et al., 2020). Then, S100β could locally modulate synaptic plasticity. In line with this idea, mutant mice lacking S100β display increased LTP and S100β level is increased following tetanization in the CA1 region of the hippocampus (Nishiyama et al., 2002; Pustylnyak et al., 2011). Further evidence from *in vivo* studies revealed that S100β overexpression is associated with impaired performance on various spatial memory tests, indicating hippocampal dysfunction (Gerlai and Roder, 1993; Gerlai et al., 1994, 1995). S100β has also been shown to support and regulate cognitive flexibility (the ability to switch between cognitively demanding tasks) in the medial prefrontal cortex (mPFC) (Brockett et al., 2018). Moreover, local infusion of S100β in the mPFC enhanced task performance. However, the mechanisms through which S100β regulates synaptic plasticity remain poorly understood. All indications are that a local reduction in [Ca^2+^]_*e*_ will significantly affect the post-synaptic Ca^2+^ elevation necessary to trigger LTP or LTD (Lisman, 1989). Still, S100β might also influence synaptic plasticity through other targets. Ionic conductance, such as the A-type current (I_*A*_) carried by the potassium channel Kv 4.2, counteract the backpropagation of AP (bAP) (Hoffman and Johnston, 1998; Goldberg et al., 2003) and its inhibition facilitate LTP (Ramakers and Storm, 2002; Watanabe et al., 2002). Interestingly, S100β has been shown to inhibit A-type current in dopaminergic neurons (Bancroft et al., 2022). On the other hand, it also increases L-type voltage-gated Ca^2+^ channels (VGCCs) which have been described as required for long-lasting synaptic plasticity (Huang and Malenka, 1993; Kim et al., 2015). Reduction of [Ca^2+^]_*e*_ by S100β release also shifts the activation threshold of NaV 1.6 channels (Morquette et al., 2015; Ryczko et al., 2021; Figure 1A) which have been shown to promote Ca^2+^ entry in dendritic spines (Stuart and Sakmann, 1995; Araya et al., 2007). More than just a simple Ca^2+^-binding protein, S100β could exert multiple antagonistic or synergistic effects on synaptic plasticity. It might even transcend this role, aligning with the concept that other gliotransmitters act as local controllers of neuronal excitability and firing patterns (Morquette et al., 2015; Ryczko et al., 2021). Its role in excitability has also been demonstrated *in vivo* as S100β-knockout mice (S100β KO) exhibit more epileptic seizures than wild-type (WT) mice (Dyck et al., 2002) and enhanced kainate-induced gamma oscillations, while the latter effect is reproduced by applications of anti-S100β antibodies in WT (Sakatani et al., 2007, 2008). Nonetheless, additional studies will be required to better understand the role of S100β and its spatiotemporal release dynamics in response to neuronal activity.

Given the importance of [K+]_*e*_ in neuronal excitability and the classical role of astrocytes as active regulators of extracellular K^+^ homeostasis, astrocytes can be inferred to exert primary control over neuronal activity. However, many studies on astrocytic signaling have primarily focused on gliotransmitters, with very few investigating extracellular K^+^ measurements and almost none examining axonal physiology, both of which are key elements in intrinsic neuronal excitability. Astrocytic [Ca^2+^]_i_ signaling has been linked to an increase in K^+^ uptake, resulting in a transient decrease in [K^+^]_o_, particularly in response to modest increases in extracellular K^+^ (5 mM), but not when K^+^ rises to pathological concentrations (Wang et al., 2012). Indeed, massive elevations of [K^+^]_o_ have been shown to cause axonal conduction failures (Meeks and Mennerick, 2004) or induce astrocyte swelling, reducing the size of the extracellular space and significantly increasing neuronal excitability (Walch et al., 2022). Under normal physiological levels of axonal activity (stimulation at 1–10 Hz), the uptake of K^+^ released by axons is mainly carried out by astroglial inward rectifier potassium channels (Kir4.1) (see Figure 1F). These receptors are important for both K^+^ uptake and redistribution back to the extracellular space for subsequent uptake by axons (Djukic et al., 2007; Bay and Butt, 2012). The importance of astroglial Kir4.1 in K^+^ clearance at higher activity frequencies (up to 20 Hz) is also crucial for signal transmission fidelity (Bay and Butt, 2012). Moreover, Kir4.1 knockout mice specifically in astrocytes exhibited seizures, indicating Kir4.1 involvement in K^+^ spatial buffering and neuronal excitability control (Djukic et al., 2007).

In addition to regulating [Ca^2+^]_*e*_ and [K^+^]_*e*_, it is important to note a new area of exploration emerging where recent studies are examining lactate transport between astrocytes and neurons. These studies show how lactate can influence excitability, synaptic plasticity (Dembitskaya et al., 2022), and neuronal activity through various mechanisms (see Cauli et al., 2023 for review).

## Gliotransmitters and STDP

Synaptic plasticity has been extensively shown to be impacted and modulated by gliotransmission (see Araque et al., 2014 for review). As demonstrated *in vivo*, selectively expressing tetanus neurotoxin (TeNT) in astrocytes to inhibit gliotransmission led to impaired behavioral and cognitive performance (Lee et al., 2014). One of the most studied, D-serine, has been extensively shown to impact LTP in the hippocampus and the cortex (Yang et al., 2003; Henneberger et al., 2010; Fossat et al., 2012). Noteworthy, D-serine is required for t-LTD at CA3-CA1 synapses and for t-LTP at hippocampal mossy fiber synapses (Rebola et al., 2011; Andrade-Talavera et al., 2016). Similarly, adenosine triphosphate (ATP) has been shown to mediate striatal t-LTP through postsynaptic A2a receptors (see Figure 1H) and to increase LTP amplitude through A1 receptors in the visual cortex (Shen et al., 2008; Bannon et al., 2017). Despite recent advances, additional studies are still required to better understand the glial control of STDP.

## Gliotransmitters and neuronal excitability

Adenosine triphosphate and glutamate can both be released from axons propagating APs or as gliotransmitters from astroglia (see Figure 1C). Glutamate released from the axonal compartment evokes only small astroglial Ca^2+^ signals directly, acting on NMDA and AMPA receptors to stimulate ATP release as a gliotransmitter, thereby indirectly inducing astroglial Ca^2+^ signals. Conversely, axonal ATP induces large Ca^2+^ signals in astrocytes, further stimulating the release of ATP and glutamate in an autocrine and paracrine manner, amplifying the axonal signal and propagating it to neighboring astrocytes (Hamilton et al., 2008). Astrocytes are highly interconnected through gap junction channels composed of connexin 30 (Cx30) and connexin 43 (Cx43), regulated by extracellular and intracellular signals that allow information exchange (see Giaume et al., 2010 for review). These astroglial networks can influence neuronal activity. One study showed that the selective elimination of Cx30 and Cx43 in astrocytes reduced the excitability of CA1 hippocampal neurons (Hösli et al., 2022), while another only removed astroglial Cx43 also observed a decrease in the excitability of orexin neurons located in the lateral hypothalamic area (Clasadonte et al., 2017). At the physiological level, astroglial ATP/adenosine release can activate axonal A_2A_ receptors, regulating both neuronal excitability and AP conduction velocity (CV) (Lezmy et al., 2021), while astroglial glutamate release can modify the AP waveform (Sasaki et al., 2011; see Figures 1A, G). Independently of neuronal activity, astrocytes also exhibit certain forms of intrinsic activity, modulating the excitability, and synchronization of neighboring neurons. For instance, it has been shown that even in the absence of neuronal activity and neuronal vesicular transmitter release, astrocytes can spontaneously release glutamate and excite adjacent pyramidal neurons in acute hippocampal slices (Angulo et al., 2004). Furthermore, slow synchronized inward currents (SIC) were observed in neighboring neurons with processes close to the gliotransmitter release source (Angulo et al., 2004). These SICs are mediated by extrasynaptic NMDA receptors mainly composed of the NR1/NR2B complex (Fellin et al., 2004; see Figure 1H). However, the current challenge lies in determining the spatial extent of this non-synaptic excitatory mechanism. Specifically, it is difficult to establish how many neurons are affected by a single event, and whether the same neuron is activated by multiple events originating from different astrocytes.

## Astrocytes, neuromodulation, and STDP

In addition to gliotransmitters, STDP is sensitive to neuromodulators such as dopamine (DA) and noradrenaline (NA), among others (see Brzosko et al., 2019 for recent review). For example, in the hippocampus, both DA and NA can extend the STDP timescale for t-LTP (Lin et al., 2003; Zhang et al., 2009). Like neurons, astrocytes have been shown to be responsive to neuromodulators. For instance, they express DA receptors, whose activation leads to changes in intracellular calcium (Corkrum and Araque, 2021). Interestingly, in the hippocampus, astrocytic D1R or D2R activation has been associated with an increase or decrease in astrocytes cytosolic calcium, respectively (Jennings et al., 2017). In the nucleus accumbens (NAc), DA-evoked astrocytes calcium signaling are associated with a decrease of excitatory synaptic transmission (Corkrum et al., 2020). However, in other brain regions, the effects on synaptic transmission and, ultimately, on synaptic plasticity remain unclear. In the hippocampus, D1R activation exerts multiple effects on t-LTP: it converts t-LTD into t-LTP, reduces t-LTP threshold, and extends t-LTP time window. In layer 5 of the prefrontal cortex, DA regulates t-LTP timing window through D1R activation and enables t-LTP through D2R activation on GABAergic interneurons to decrease inhibition. It is unknown whether astrocytes are partially or fully involved in some of these effects. Furthermore, adding to the complexity, it has been reported that astrocytes in the prefrontal cortex (PFC) respond to dopamine (DA) via α-adrenergic receptors (α-ARs), resulting in the release of ATP and likely regulating synaptic transmission (Fellin et al., 2004; Pittolo et al., 2022). The same receptor is also responsive to NA which triggers as well astrocytic calcium increases (Paukert et al., 2014; Slezak et al., 2019; Oe et al., 2020). In the visual cortex, α-ARs activation promotes an LTD-only STDP but the coactivation of α-ARs and β-ARs allows the conventional bidirectional STDP curve (Huang et al., 2013, 2014). β-ARs activation alone is sufficient to induce a robust t-LTP (Seol et al., 2007). Astrocytes express and respond as well to β-ARs activation through intracellular calcium increases (Oe et al., 2020). Interestingly, β-ARs activation has been shown to inhibit A-type current which could explain the effect on t-LTP (Yuan et al., 2002). In astrocytes, α-ARs activation has been linked to increased glutamate uptake (Hansson and Rönnbäck, 1989, 1992), reduced [K+]_*e*_ clearance (Åkerman et al., 1988; Wotton et al., 2020) and even secretion of brain-derived neurotrophic factor (BDNF) (Juric et al., 2008; Koppel et al., 2018). On the contrary, β-ARs activation has been linked to increased GABA uptake (Hansson and Rönnbäck, 1989, 1992), increased [K+]_*e*_ clearance (Wotton et al., 2020) and higher BDNF secretion as well. Interestingly, similar to what is observed in neurons, the level of NA exerts antagonistic effects because α-ARs have highest affinity compared to β-ARs. In the hippocampus, t-LTP can be BDNF-dependent or -independent depending respectively on high or low postsynaptic activities (Edelmann et al., 2015). This discussion could be extended to other neuromodulators such as acetylcholine or serotonin which are known to affect astrocytes calcium dynamics (Pacholko et al., 2020) and have been shown to greatly influence STDP (Seol et al., 2007; Brzosko et al., 2017). This underscores the importance of not overlooking astrocytes when studying the effects of neuromodulation on synaptic plasticity. It also invites a reconsideration of previous findings. The dynamics of neuromodulation and their impact on synaptic plasticity should be further explored within the tripartite synapse model.

## Astrocytes and axonal plasticity

Hildebrand (1971), based on electron microscopy studies in the feline spinal cord, demonstrated that certain astrocytic processes are closely located at the nodes of Ranvier. Subsequently, these observations were confirmed in other animals in the following years (Waxman and Swadlow, 1976; Berthold and Carlstedt, 1977; Sims et al., 1985). These authors named these glial cells as perinodal astrocytes. Other quantitative measurements observed that the axon initial segment (AIS) of Purkinje cells is densely covered by glial processes, covering 74.2% of the total surface in cats and 85.6% in rats (Somogyu and Hámori, 1976). More recently, with the help of more advanced technology, it was confirmed that these glial processes were astrocytic (Iwakura et al., 2012). Additionally, it has also been revealed that the AIS of ganglion cells in the optic nerve receive contacts mainly from astrocytic processes (Stone et al., 1995). Today, it is widely accepted that the AIS and the nodes of Ranvier, far from being rigid structures, can undergo remodeling depending on neuronal activity. Several reviews have been published on experimental findings demonstrating the structural plasticity of these excitable axonal domains and their functional implications (see Grubb et al., 2011; Susuki and Kuba, 2016; Yamada and Kuba, 2016; Petersen et al., 2017 for review). However, the field remains immature, and periaxonal astrocytes have barely been included as key agents in this type of axonal plasticity. The first to do so was the group of Douglas Fields, pioneers in demonstrating how myelin-forming glial cells can detect neuronal impulses to optimize the speed and synchrony of information. They unexpectedly detected a link between astrocytes, myelination, and neuronal activity. They reported that ATP released by DRG neuron axons firing APs induces the release of leukemia inhibitory factor (LIF) by astrocytes, which in turn promotes myelination in late stages, beyond development (Ishibashi et al., 2006; see Figure 1D). This discovery shed light on existing hints of a possible link between astrocytic function and myelin structure, in GFAP-deficient mice that exhibited abnormalities in myelination (Liedtke et al., 1996). More recently, this same group provided a mechanism involving perinodal astrocytes as responsible for remodeling the myelin sheath and nodal structure. Specifically, perinodal astrocytes regulate myelin attachment to the axon through vesicular release of thrombin protease inhibitors, just at the binding site of neurofascin 155 (NF155) and Caspr1, interfering with the detachment of outer paranodal loops and widening of the nodal space (Dutta et al., 2018; see Figure 1B). These anatomical changes can have electrophysiological and functional consequences if astrocytes do not prevent thrombin-dependent NF155 (axoglial contact) cleavage. In this case, widening of the nodal space allowed lateral diffusion of voltage-gated Na^+^ channels and slowed down the CV of the AP. Additionally, it is noteworthy that neural AIS excitability was altered, and CV of APs in the nodes of Ranvier was also reduced when periaxonal astrocytes responded to the activity of myelinated neurons in layer 5 of the cerebral cortex by releasing vesicles containing ATP, subsequently converted into adenosine (Lezmy et al., 2021; see Figure 1). Consequently, these findings suggest that perinodal astrocytes could contribute to establishing appropriate myelin thickness and nodal properties essential for optimizing conduction latency in neuronal circuits. However, their involvement in AIS remodeling remains largely unexplored.

## Astrocytes and oligodendrocytes: new pathways in axonal plasticity

Accumulating evidence indicates that astrocytes may be coupled to oligodendrocytes via gap junctions, forming extensive syncytia known as panglial networks (see Stephan et al., 2021 for review). As mentioned previously, astrocytes can express Cx30 and Cx43, whereas oligodendrocytes express different connexins, namely Cx47, Cx32, and Cx29, with Cx29 contributing minimally to intercellular communication (Altevogt et al., 2002; Nagy et al., 2003). Therefore, astrocyte–oligodendrocyte (A/O) coupling might predominantly be mediated by heterotypic channels: Cx43/Cx47, Cx30/Cx32, Cx43/Cx32, or Cx30/Cx47 (Orthmann-Murphy et al., 2007; Griemsmann et al., 2015). The primary function attributed to panglial networks is the modulation of neuronal excitability through the spatial buffering of K^+^ (Kamasawa et al., 2005; Battefeld et al., 2016). Similar to astrocytes, oligodendrocytes can detect and respond to axonal activity, either via the release of axonal K^+^ (Kamasawa et al., 2005; Looser et al., 2024) or through vesicular release of axonal glutamate (Micu et al., 2016; see Figures 1E, F). In the first case, following an AP, the pathway for axonal K^+^ flow not only exits the nodes of Ranvier toward the astrocytic endfeet, as was initially thought when myelin was considered to play a passive role (Waxman and Black, 1984), but predominantly occurs in the juxtaparanodal axolemma, the area enclosed within the myelin sheaths. Once released, the influx of K^+^ is transported then to the astrocytic syncytium, first through oligodendroglial Kir4.1 channels (Looser et al., 2024), then crossing successive paranodal loops, and finally through A/O junctions. This is presumably driven by an electrochemical force due to the high difference in electrical potential (+40 to +74 mV in the internodal compartment versus −85 mV in the coupled astrocytic syncytium). Another portion of the axonal K^+^ influx returns to the axon (Kamasawa et al., 2005). In the second case, high-resolution two-photon microscopy has shown that APs induce vesicular release of axonal glutamate, activating internodal NMDA receptors (containing GluN2D and GluN3A) and leading to an increase in intracellular Ca^2+^ in oligodendrocytes (Micu et al., 2016). Several studies have demonstrated that these axon-oligodendrocyte-astrocyte signaling mechanisms can produce biophysical changes in the fine structure of myelin, as well as modulate neuronal excitability and axonal conduction (Yamazaki et al., 2010; Battefeld et al., 2016; Micu et al., 2016). Additionally, there has been speculation that certain abnormalities in these intercellular interactions may trigger pathologies such as schizophrenia, multiple sclerosis, or epilepsy (Lundgaard et al., 2013; Bedner et al., 2015; Micu et al., 2016). Despite these promising advances, much work remains to be done for identifying which elements can locally modulate axonal plasticity for physiological purposes and which may play a more pathological role.

## Astrocytes in others forms of plasticity

While our review focuses on astrocyte interactions with synaptic plasticity (STDP) and intrinsic plasticity, it is important to note that astrocytes also play roles in other forms of plasticity, such as homeostatic plasticity and synaptic scaling, both crucial for maintaining brain activity homeostasis in response to prolonged internal or external disturbances and ensuring a proper excitatory–inhibitory (E/I) balance (Turrigiano, 2007; Perez-Catalan et al., 2021). Several gliotransmitters, including SPARC (secreted protein acidic and rich in cysteine) and the cytokine IL-33, have been implicated in normal synaptic scaling. Both are essential for enhancing activity following deprivation induced by TTX (Jones et al., 2011; Wang et al., 2021). Considerable research has also focused on tumor necrosis factor α (TNF-α), whose secretion is essential for synaptic scaling (Stellwagen and Malenka, 2006; Heir and Stellwagen, 2020). In hippocampal cultures, astrocytes—but not microglia—produce TNF-α to support homeostatic plasticity in response to chronic TTX treatment (Heir et al., 2024). Additionally, astrocytes contribute to maintaining network homeostasis by participating in activity-dependent synapse remodeling, which involves both the elimination and formation of synapses through various mechanisms, as recently reviewed by Kim and Chung (2023).

## *In vivo* consideration

Although this review primarily focused on *in vitro* studies, a significant amount of *in vivo* research was also conducted, particularly regarding the role of astrocytes in brain rhythms. Logically, since brain oscillations arise from synchronized neuronal activity, the previously described effect of astrocytes could influence them. In line with this idea, gliotransmitters have been associated to the modulation of brain rhythms. For instance, in a mouse model with impaired astrocyte-specific exocytosis, theta synchronization between the dorsal hippocampus and prefrontal cortex was disrupted, resulting in decreased performance in spatial learning and reference memory (Sardinha et al., 2017). In contrast, gamma activity in the somatosensory cortex is enhanced in a mouse model with impaired astrocyte calcium signaling, while it decreases during the pharmacological activation of astrocytes (Lines et al., 2020). Specifically, the genetic ablation of GABA_*B*_ receptors on astrocytes resulted in a decline in decision-making performance and a reduction in low-gamma power (Mederos et al., 2021). Here, we focused on astrocytes, but it is worth noting that oligodendrocytes, through the modulation of myelin sheaths, and microglia, through ion buffering, also play important roles in modulating brain oscillations (Lewen et al., 2020; Dubey et al., 2022). The role of glial cells in brain rhythms and their implications for memory storage and processing in health and diseases have been excellently reviewed recently (Phatnani and Maniatis, 2015; Andrade-Talavera et al., 2023; Brandebura et al., 2023).

## Conclusion

The role of astrocytes in various forms of plasticity is only beginning to be understood, and there is much more to uncover. Previous studies could be revisited to reassess the potential role of astrocytes, and future research should always consider their involvement. From synaptic to intrinsic or even axonal plasticity, astrocytes appear to have a significant influence (see Figure 1). Our discussion could extend to other forms, such as homeostatic plasticity, and to other glial cells, such as oligodendrocytes, which we briefly mentioned. One thing is certain: ideas, projects, and experiments will never cease for those intrigued by neuron–glia communication, as it now appears that they are not mere conjectures, nor are we so unequipped to fathom the function of glial cells, echoing the sentiments of Santiago Ramón y Cajal over a century ago.
